# Efficacy of a Graphene Oxide/Chitosan Sponge for Removal of Radioactive Iodine-131 from Aqueous Solutions

**DOI:** 10.3390/life11070721

**Published:** 2021-07-20

**Authors:** Tanate Suksompong, Sirikanjana Thongmee, Wanwisa Sudprasert

**Affiliations:** 1 Department of Physics, Faculty of Science, Kasetsart University, Bangkok 10900, Thailand; tanate.su@ku.th (T.S.); fscisjn@ku.ac.th (S.T.); 2 Department of Applied Radiation and Isotopes, Faculty of Science, Kasetsart University, Bangkok 10900, Thailand

**Keywords:** graphene oxide/chitosan sponge, adsorbent, adsorption isotherm, iodine-131, radioactive waste management

## Abstract

Iodine-131 is increasingly used for diagnostic and therapeutic applications. The excretion of radioactive iodine is primarily through the urine. The safe disposal of radioactive waste is an important component of overall hospital waste management. This study investigated the feasibility of using graphene oxide/chitosan (GO/CS) sponges as an adsorbent for the removal of iodine-131 from aqueous solutions. The adsorption efficiency was investigated using iodine-131 radioisotopes to confirm the results in conjunction with stable isotopes. The results revealed that the synthetic structure consists of randomly connected GO sheets without overlapping layers. The equilibrium adsorption data fitted well with the Langmuir model. The separation factor (R_L_) value was in the range of 0–1, confirming the favorable uptake of the iodide on the GO/CS sponge. The maximum adsorption capacity of iodine-131 by GO/CS sponges was 0.263 MBq/mg. The highest removal efficiency was 92.6% at pH 7.2 ± 0.2. Due to its attractive characteristics, including its low cost, the ease of obtaining it, and its eco-friendly properties, the developed GO/CS sponge could be used as an alternative adsorbent for removing radioiodine from wastewater.

## 1. Introduction

Iodine is one of the best-known elements because it is an essential mineral for the body. Iodine, which enters the body with food, binds to the amino acid tyrosine and forms a hormone called thyroxin in the follicular cells of the thyroid gland. On the other hand, if the body is exposed to radioactive iodine, such as iodine-131, it can be dangerous. A considerable body of evidence from research has shown the serious effects of iodine-131 exposure on the risk of thyroid cancer [1,2]. Recent research by Zupunski et al. [3] found a correlation between subjects exposed to iodine-131 from Chernobyl’s fallout during childhood at age ≤18 years and thyroid cancer risk. This is of great concern, as iodine-131 is routinely administered as a radiopharmaceutical in nuclear medicine in the form of sodium iodide (NaI) for diagnosis and treatment of thyroid disease [4]. Approximately 80% of the administered iodine-131 is excreted in the urine [5]; therefore, it has potential to be discharged to sewage wastewater if not carefully controlled. Elevated concentrations of iodine-131 have been observed in municipal wastewater treatment plants following the application of therapeutic doses [6]. Rose and Swanson [7] found iodine-131 in sewage sludge from three water pollution control plants, ranging from 0.027 ± 0.002 to 148 ± 4 Bq/g dry weight.

The harmful effects of iodine-131 are gaining more attention around the world as an increase in iodine-131 contamination has been detected in the environment. Recently, iodine-131 has been detected in river water and aquatic organisms in South Korea. This indicates that radioactive iodine-131 can enter the environment through wastewater treatment plants through increased medical use [8]. Additionally, in 2020, Mosos et al. [9] reported the results of iodine-131 measurements at a wastewater treatment plant in Bogota, Colombia, and found the highest activity in raw water in the morning each day. The concentration of iodine-131 exceeds the reference value for drinking water and is close to the discharge limit in water bodies in Columbia. This indicates the risk of iodine-131 contamination of the environment from medical activities. The United States Environmental Protection Agency (USEPA) has established drinking water standards that specify no more than 4 millirems of beta radiation per year, which is equivalent to iodine-131 activity of not more than 3.0 pCi/L or 0.111 Bq/L [10].

The World Health Organization (WHO) has issued guidance on the level of iodine-131 that can be released into water bodies through sewage effluent: not more than 10 Bq/L [11]. In general, a waste collection system must be designed to store radioactive waste over a period of time. However, as the number of patients increases, many older facilities have limited space for waste storage. Therefore, they are unable to develop new systems, or these systems require large investments. With respect to this, if iodine-131-adsorbing materials can be developed, they will reduce the radioactivity of the radioactive waste released into the sewage waste drainage system. The use of absorbents can significantly reduce the volume of radioactive waste destined for temporary decay storage. This development is very beneficial for the safety of nearby personnel and the environment. In this research, we synthesized GO/CS sponges, a material with the potential to absorb or separate iodine-131 from water, which could be used in the future for radioactive waste management in hospitals. 

## 2. Materials and Methods

### 2.1. Materials

Chitosan was obtained from Hunan Insen Biotech Co., Ltd in China (molecular weight: 10,000–20,000 Daltons). Graphite flakes were procured from Jiangsu XfNano materials Tech Co., Ltd, China, with a mean size of 100 mesh and a purity of 99.5% (model number XF051). Sulfuric acid (H_2_SO_4_, 95.0–98.0%) was obtained from Ajax Finechem (Australia); potassium permanganate (KMnO_4_, ≥99.0%), phosphoric acid (H_3_PO_4_, ≥99.0%), sodium nitrate (NaNO_3_, ≥99.0%), sodium hydroxide (NaOH, ≥99.0%), sodium iodide (NaI, ≥99.0%), and ethanol (C_2_H_5_OH, ≥99.9%) were obtained from Merck (Kenilworth, NJ, USA). Hydrochloric acid (HCl, ≥99.0%) and hydrogen peroxide (H_2_O_2_, 30%) were obtained from Sigma Aldrich (St. Louis, MO, USA).

### 2.2. Synthesis of Graphene Oxide Suspension

GO was prepared using a modified Hummer’s method [12]. Briefly, graphite flakes (3.0 g) were mixed with KMnO_4_ (9.0 g); then, the mixture of H_2_SO_4_ (360 mL) and H_3_PO_4_ (40 mL) was slowly added under continuous stirring in a water bath and stirred for 15 min. Next, 3.0 g of NaNO_3_ was added to the solution, followed by 400 mL of deionized water. This mixture was magnetically stirred for 4 h. After that, 30 mL of H_2_O_2_ was added to the solution. The filtrate obtained was washed with 200 mL of HCl (10%), then 200 mL of ethanol was added and the suspension was stirred at room temperature for 60 h. The GO was washed with 5% HCl and DI water until a neutral pH was achieved. Subsequently, the suspension was kept without disturbance around 3–4 h until the GO settled on the bottom of the beaker and the volume of the suspension had decreased to 250 mL. The final concentration of the GO suspension was 12.0 mg/mL.

### 2.3. Preparation of GO/CS Sponges

CS was prepared by dissolving 6.0 g of chitosan in 294 mL of acetic acid (2.0%). The GO suspension and the CS solution were mixed at a ratio of 2:1. The obtained solution of GO/CS was sonicated for an additional 1 h and aged for 12 h to form a homogeneous GO/CS suspension [13]. The final concentration of the stock GO/CS suspension was 0.01467 mg/µL. Preparation of the GO/CS sponges was started by pouring the GO/CS suspension into a Teflon cup before freezing it at −20 °C, followed by freeze-drying (vacuum of 0.1 mbar for 24 h), then baking it at 60 °C for 48 h to eliminate moisture.

### 2.4. Characterization of GO/CS Sponges

The GO/CS sponge samples were characterized by different techniques. Scanning electron microscopy (SEM; Quanta model 450) was used to observe the surface morphology of the GO and the GO/CS suspension. Raman spectra were obtained with a DXR Raman microscope (aperture: 50 micrometer slit; laser spot size: approximately 3.1 micrometers; power: 10 mW) with a 532 nm laser. The functional groups on the adsorbent samples were investigated with a Fourier-transform infrared spectrometer (FT-IR; Perkin Elmer, Spectrum model).

### 2.5. Adsorption Experiments

The adsorption experiments were carried out by using the stable isotope of iodine-127 and the radioactive isotope of iodine-131. As the stable and radioactive iodine isotopes have similar chemical properties, the stable isotope was mainly used to perform all the adsorption experiments except for the adsorption ability test in order to avoid radiation exposure and radioactive contamination of the devices and instruments. In addition, the adsorption efficiency of GO/CS for removing iodine-131 was easily determined by radioactivity analysis using gamma spectrometry, rather than indirect analysis by measuring the iodine concentration with other instruments.

The adsorption capacity of the GO/CS sponge was determined using absorption techniques with a UV–vis spectrophotometer (SHIMADZU UV-2600). The iodide adsorption spectra were observed at 226 nm [14]. The calibration curve for the relationship between the concentration of the NaI solution (0.1 to 4.0 mg/L) and its absorbance was established. The adsorption capacity at various iodine concentration was determined with 2.0 mg of GO/CS sponges mixed in a 50 mL NaI solution at concentrations of 0.1–4 mg/L. Next, the mixture was agitated in a shaker at room temperature for 24 h. The GO/CS sponges were separated from the solution using glass microfiber filters with a 0.2 µm pore size. The concentration of iodide in the remaining solution was measured with a UV–vis spectrophotometer, and the equilibrium adsorption amounts, q_e_ (mg/g), were calculated via Equation (1) [15].

(1) $$q_{e}=\frac{\left(C_{i}-C_{t}\right)V}{m}$$

where *C_i_* (mg/L) and *C_t_* (mg/L) are the initial sodium iodide concentration and the concentration at any time *t* (min), respectively; *V* (mL) is the volume of the sodium iodide solution; and *m* (g) is the mass of the CS/GO sponges.

The effects of the parameters, including adsorbent dosage, contact temperature, contact time, and pH, on the removal of iodide were studied. To explore the effects of adsorbent dosage, the GO/CS sponges (1.5–6.0 mg) were mixed in a 50 mL NaI solution (1.5 mg/L) for 2 h. The mixture was adjusted to pH 7.2 ± 0.2. Afterwards, the GO/CS sponges were filtered, and the residual concentration of the iodide in the supernatant was estimated spectrophotometrically using the established calibration curve. The adsorption capacity at equilibrium (q_e_) was calculated from Equation (1). The adsorption capacity test at different temperatures was conducted by mixing 2.0 mg of GO/CS sponges with a 50 mL NaI solution (1.5 mg/L) at pH 7.2 ± 0.2 for 2 h. The mixing temperature was varied from 5 to 45 °C. The q_e_ at each temperature was determined as previously described. The effect of contact time on the adsorption capacity was studied by setting up the experiment as above and then varying the contact time from 5 min to 24 h at room temperature. The influence of the solution’s pH on the adsorption capacity was evaluated by adjusting the pH of solution with HCl/NaOH solution to pH values of 4 to 9.

The adsorption isotherm experiment was conducted by adding 2.0 mg of CS/GO sponges into a 50 mL NaI solution at different concentrations (0.5, 1.0, 1.5, 2.0, and 4.0 mg/L). The adsorption data were fitted with the Langmuir and Freundlich models [16,17] as presented in Equations (2) and (3), respectively.

(2) $$\mathrm{Langmuir}\;\mathrm{model}:\frac{C_{e}}{q_{e}}=\frac{1}{K_{L}\;q_{max}}+\frac{C_{e}}{q_{max}}$$

where *C_e_* (mg/L) is the concentration of NaI at equilibrium, *q_e_* (mg/g) is the amount of adsorbed sodium iodide on the surface of the GO/CS sponges at equilibrium, *K_L_* (L/mg) is the Langmuir constant related to the adsorption capacity, and *q_max_* (mg/g) is the maximum adsorption capacity of the GO/CS sponges.

(3) $$\mathrm{Freundlich}\;\mathrm{model}:\;\log\;q_{e}=\log K_{F}+\left(\frac{1}{n}\right)logC_{e}$$

where *K_F_* (mg/g) and 1/*n* are Freundlich constants representing the coefficient and intensity of adsorption, respectively. 

The removal efficiency of iodine-131 by GO/CS was determined. A stock solution of iodine-131 in NaI with a radioactive concentration of 11.84 MBq/mL was obtained from the Thailand Institute of Nuclear Technology (Public Organization). It was diluted with deionized water until a final concentration of 140.78 kBq/mL was reached. The adsorption experiments were carried out in 50 mL plastic tubes to prevent the adsorption of radionuclides onto the glass wall. The experiment was conducted to explore the optimum equilibrium time by varying the incubation period from 0.5 to 48 h. The 30 mL iodine-131 solutions with 4.223 MBq of activity were added into each tube, which contained GO/CS sponges at the same concentration in each tube. The mixtures were then stirred for 10 min and allowed to equilibrate for 0.5 to 48 h at room temperature. The GO/CS suspension was filtered out by glass microfiber filters with a pore size of 0.2 µm. Subsequently, the radioactivity of the iodine-131 in the solution was measured with a gamma spectrometer (Canberra, model DSA 1000) equipped with a coaxial HPGe detector (model GR2519) 51.7 mm in diameter and 58.5 mm in length. This system delivered a resolution of 1.82 keV for 1332.5 keV gamma rays from cobalt-60, with an efficiency of 0.7011% for 364 keV gamma rays of iodine-131 calculated using the standard source (Eckert & Ziegler source No. 1868-30) with energies in the range of 88–1836 keV. Gamma spectrum analysis was performed using Genie 2000 software. The radioactivity of iodine-131 [18], given in Becquerel (Bq), was calculated using Equation (4):

(4) $$A=\frac{NC_{e}}{t\varepsilon pV}$$

where A is the activity concentration of iodine-131 (Bq/L), *N* is the number of counts in the photo-peak at an energy level of 364 keV, *C_e_* is the true coincidence summing correction factor, *t* is the measurement time (s), *ε* is the detector’s efficiency, *p* is the probability of disintegration of the radionuclide, and *V* is the volume of the solution (L).

The removal efficiency of iodine-131 by GO/CS was determined by Equation (5):

(5) $$\mathrm{Removal}\;\mathrm{efficiency}\;\left(\%\right)=\left[\frac{A_{0}-A_{1}}{A_{0}}\right]\times 100$$

where *A*_0_ is the initial radioactivity of iodine-131 before GO/CS adsorption and *A*_1_ is the radioactivity of the resulting supernatant. 

## 3. Results and Discussion

### 3.1. Characterization of GO/CS Sponges

**Figure 1:**
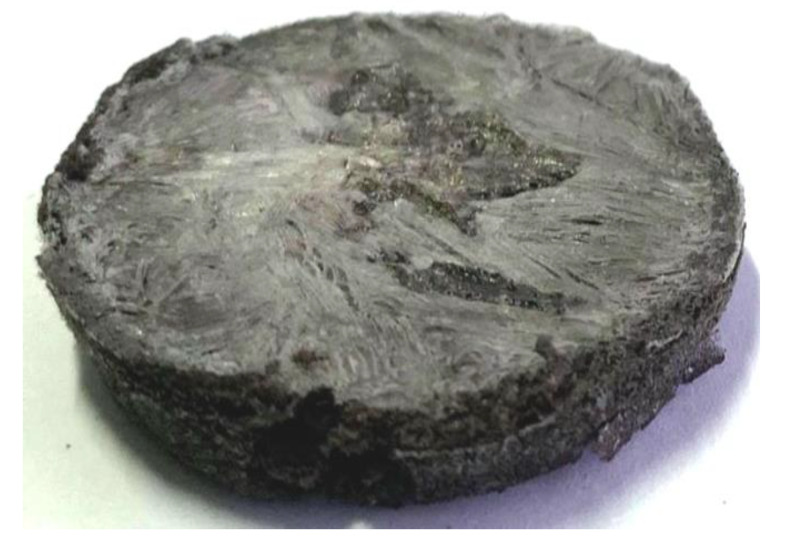
A GO/CS sponge after the freeze-drying process.

GO/CS sponges with a proportion of 2:1 (v/v) were generated by a freeze-drying process at −20 °C. The shape was similar to the Teflon cup. Their physical characteristics were solid and light with a three-dimensional structure, as shown in Figure 1.

**Figure 2:**
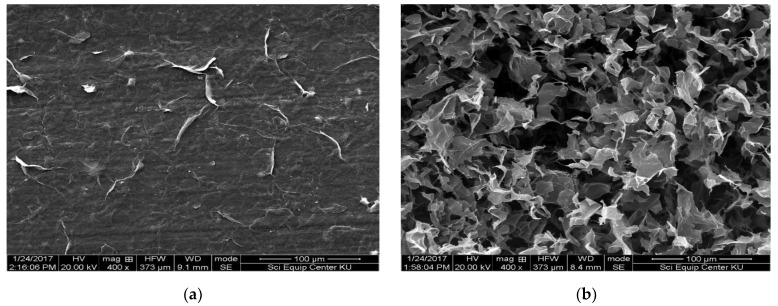

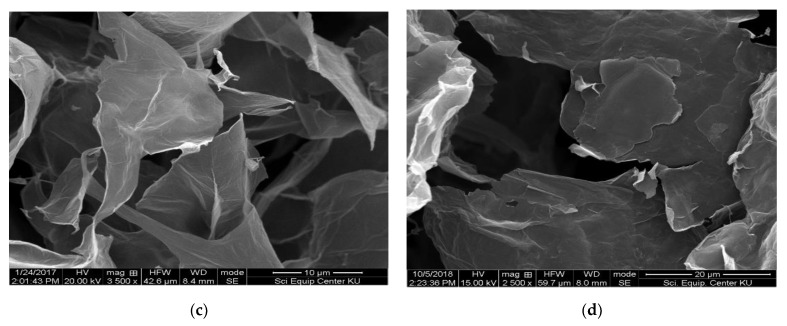
SEM images of (**a**) GO and (**b**–**d**) GO/CS sponge at different magnifications.

SEM images were used to determine the morphology of GO and GO/CS sponges. Figure 2a shows the GO, revealing that the surface of GO is generally smooth, whereas the edges of the GO/CS sponge are raised and bent, as seen in Figure 2b. This may be due to the different structure and hardness between CS and GO. It was proposed that the adequate dispersion of GO in CS was achieved through the interactions between the amino groups (-NH_2_) in CS and the carboxylic groups (-COOH) in GO [19,20]. Moreover, Figure 2c shows that the GO sheets lined up randomly and the GO sheets connected together without any overlay, resulting in gaps. In Figure 2d, the gaps inside the GO/CS sponge’s structure beneath the surface were caused by air that replaced the liquid during the freeze-drying process. This demonstrates that the internal structure of the GO/CS sponge was full of a GO sheet network.

**Figure 3:**
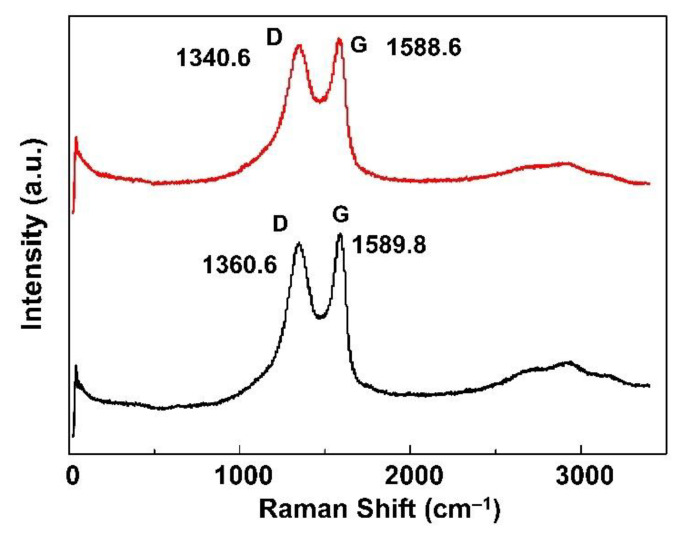
Raman spectra of GO and GO/CS sponges.

To observe the ordered and disordered crystal structures of GO and GO/CS, Raman spectroscopy was used. Figure 3 shows the Raman spectra of GO and GO/CS sponges. We can see that the results of these two samples are similar. The Raman spectrum of GO shows two characteristic peaks at 1340 cm^−1^ (D-band, C-C) and 1588 cm^−1^ (G-band, C=C). The D-band is caused by the breathing modes of carbon atoms (six-atom rings) and the G-band corresponds to the high-frequency E_2g_ mode of sp^2^-hybridized carbon atoms [21]. The D-band to G-band intensity ratio (I_D_/I_G_) for GO was 0.8032. For the GO/CS suspension, shifts at the D-bands (20 cm^−1^) and G-bands (2 cm^−1^) were observed compared with GO. The size of the I_D_/I_G_ ratio of GO/CS (0.933) was slightly greater than the I_D_/I_G_ ratio of GO (0.8032). This result suggests that the structure of the GO/CS suspensions was similar to that of ordered carbon nano-sheets [22]. 

**Figure 4:**
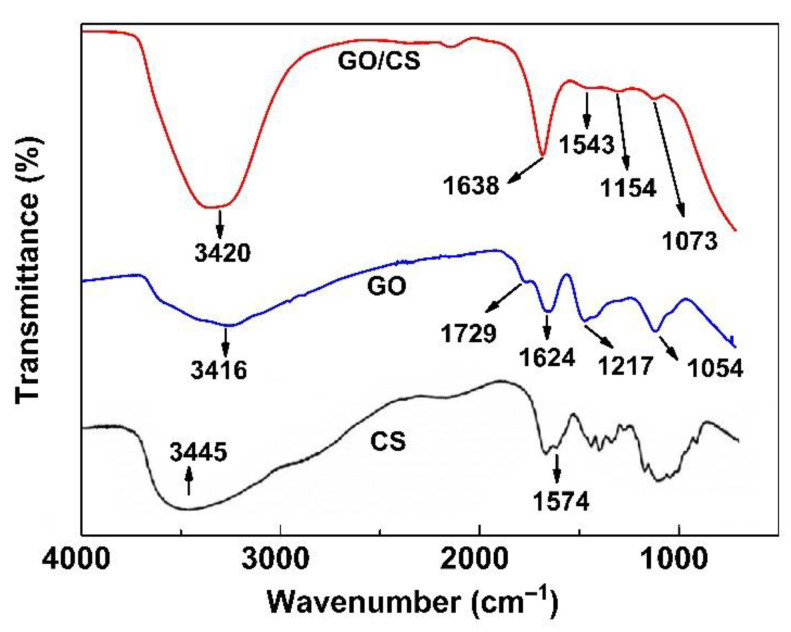
FT-IR spectra of GO, CS, and GO/CS.

**Figure 5:**
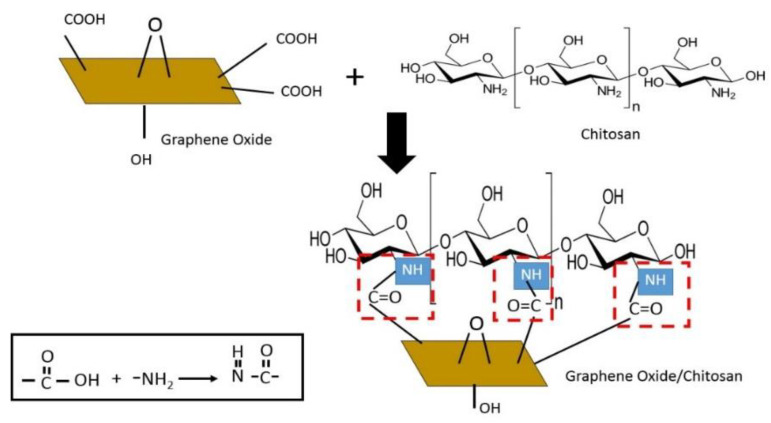
Proposed mechanism of the reaction between GO and CS to form the GO/CS suspension.

The functional groups of GO, CS, and GO/CS were investigated by FT-IR, as shown in Figure 4. The GO spectrum showed adsorption peaks that were assigned to the functional group of O-H bonds at 3416 cm^−1^, the C=O stretches of the carboxylic group (1729 cm^−1^), the stretching vibrations of aromatics (C=C) at 1624 cm^−1^, epoxy C−O−C stretches at 1217 cm^−1^, and alkoxy C−O bonds at 1054 cm^−1^. Our results were similar to the results reported by Liu et al. [23]. The CS spectrum showed the main adsorption peak of the NH_2_ group of CS at 1574 cm^−1^. In the case of the GO/CS suspension, the FT-IR spectrum showed a new adsorption peak at 1638 cm^−1^ related to amide (N-H), whereas the C=O at 1729 cm^−1^ disappeared. This might have been caused by the NH_2_ of CS reacting with the C=O of GO to form the functional group of N-H in the GO/CS suspension. Moreover, the C−O in the spectrum of GO/CS at 1073 cm^−1^ was less intense than that in the spectrum of GO at 1054 cm^−1^. This is because the CS interacted with the OH groups of GO [24]. A diagram of the mechanism of the GO/CS suspension from the reaction between GO and CS is displayed in Figure 5. This reaction scheme explains that, when GO is mixed with the CS solution, electrostatic interactions are formed between the C=O group of GO and the N-H groups of CS, resulting in the stable GO/CS composite with improved elasticity [24].

### 3.2. Adsorption Capacity

**Figure 6:**
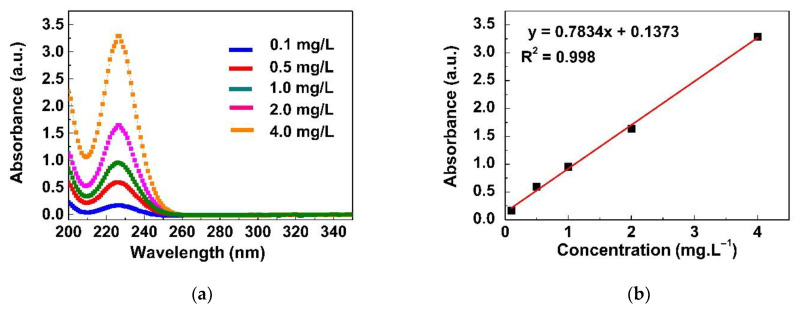
(**a**) UV–visible absorption spectra of NaI at concentrations of 0.1–4.0 mg/L at 25 °C and pH 7.2 ± 0.2; (**b**) calibration curve used for determination of the NaI concentration.

A series of experiments were conducted to determine the effect of adsorbent concentration, temperature, contact time, and pH on the adsorption rate. UV–vis spectroscopy was used to observe the concentration of NaI remaining in the supernatant after adsorption by the GO/CS sponges. The UV–vis absorption spectra of different NaI concentrations are shown in Figure 6a. The maximum absorption was found at a wavelength of 226 nm [25]. A calibration curve was established to determine the concentration of NaI derived from the absorbance by using the regression equation y = 0.7834x + 0.1373, as shown in Figure 6b. 

**Figure 7:**
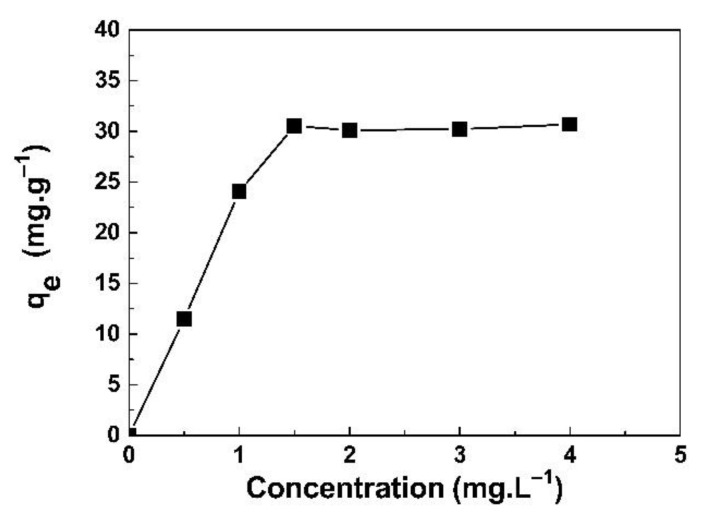
The NaI adsorption capacity (q_e_) of 2.0 mg of GO/CS sponges with a contact time of 24 h at a pH of 7.2 ± 0.2 and room temperature.

Figure 7 shows the adsorption capacity results for 2.0 mg of the GO/CS sponges mixed with the NaI solution at concentrations of 0.1–4 mg/L. The adsorption capacity at equilibrium (q_e_) did not change in the 30 mg/g range, and the highest q_e_ was found at 30.52 mg/g with a concentration of 1.5 mg/L. This was probably due to the surface of the GO/CS sponge being limited and thus insufficient to adsorb NaI from the solution. 

**Figure 8:**
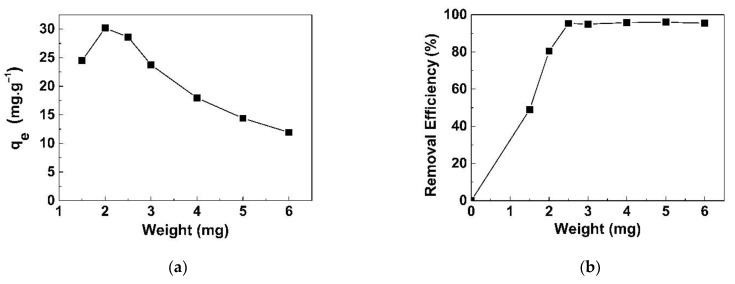
Effect of adsorbent dosage on NaI adsorption onto GO/CS sponges: (**a**) q_e_ and (**b**) removal percentage at a concentration of 1.5 mg/L at 25 °C, pH 7.2 ± 0.2.

The effect of adsorbent dosage on adsorption capacity was investigated in the range of 1.5–6.0 mg of GO/CS sponges. The q_e_ and removal percentage of NaI were plotted against dosage, as illustrated in Figure 8a,b, respectively. The q_e_ increased as the adsorbent dose increased from 1.5 to 2.0 mg, then decreased from 2.0 mg onward. The highest q_e_ was found at 30.17 mg/g, whereas the lowest q_e_ was found at 11.9 mg/g. The removal percentage increased rapidly from 45 to 95% at 1.5–2.5 mg, and it tended to be stable at approximately 95%.

**Figure 9:**
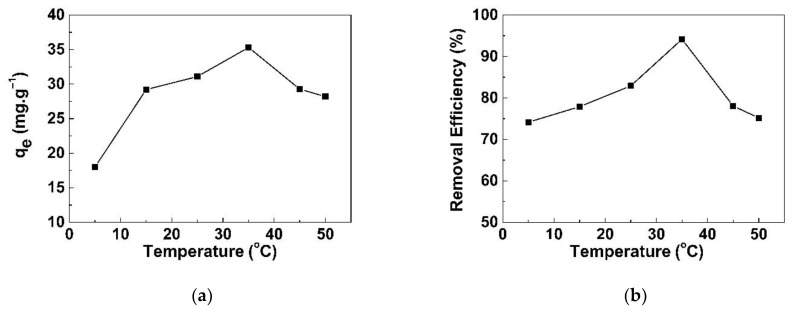
Effect of temperature on NaI adsorption onto GO/CS sponges: (**a**) q_e_ and (**b**) percentage removal at a concentration of 1.5 mg/L at pH 7.2 ± 0.2.

The effect of temperature on adsorption capacity was investigated from 5 to 50 °C. The adsorption capacity increased with the increase in temperature from 5 to 35 °C then decreased until the temperature was 50 °C, as shown in Figure 9a. The highest removal was found at 95% (Figure 9b), which was equivalent to 35.3 mg/g at 35 °C. This observation might be explained by the iodide ions (I^−^) moving and reacting with hydrogen when the temperature increased from 5 °C to 35 °C; after that, the adsorption decreased because the physical adsorption mechanism between the -NH_3_^+^ and I^−^ functional groups on the GO/CS sponge was an exothermic process. The higher the temperature, the lower the adsorption capacity (30.1 mg/g). These results, according to studies by Besemer et al. [26], suggest that iodide ions react with hydrogen on the surface of an adsorbent, resulting in two types of hydrogen bonds, namely O···H–O–H···I and I^−^···H–O–H···I^−^. For implementation, it might be more practical to use room temperature (25 °C) rather than 35 °C. This is because of the small difference in adsorption capacity found between these two temperatures. 

**Figure 10:**
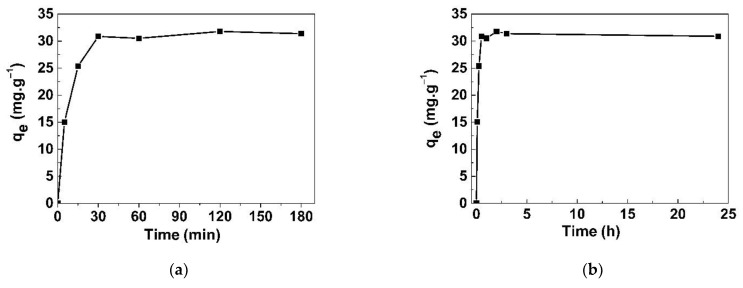
Effect of contact time on NaI adsorption onto GO/CS sponges: (**a**) 5 min to 180 min and (**b**) 5 min to 24 h.

The effects of contact time from 5 to 180 min and from 5 min to 24 h on the adsorption capacity are shown in Figure 10a,b, respectively. The q_e_ rapidly increased during the first 30 min as the adsorbent still had a large surface, then it slowly increased until it became stable when the adsorption reached the saturation point. From 30 min to 24 h, the q_e_ was relatively stable in the range of 30.8–31.8 mg/g.

**Figure 11:**
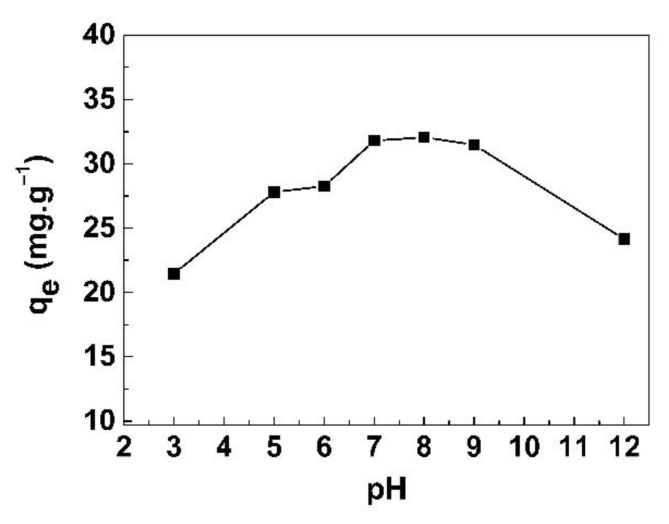
Iodide ion adsorption capacity of GO/CS sponges at pH 3 to pH 12.

To explore the effects of pH on the adsorption capacity, the pH of the mixing solution was varied from 3 to 12. Figure 11 shows the adsorption capacity of the GO/CS sponges for iodide ions at pH 3 to pH 12. The results show that the lowest adsorption was found at pH 3 and adsorption increased until pH 8. After that, the adsorption continuously decreased from pH 9 to pH 12. This may be because the capabilities of H^+^ and I^−^ can be combined in the form of hydrogen iodide (HI). Normally, at a low pH [27], the -NH_2_ group of CS will change to −NH_3_^+^, resulting in its binding to I^–^. However, when GO was mixed with CS, the -NH_2_ groups in CS bound with GO instead of I^−^, and only GO could adsorb I^−^, resulting in less adsorption at low pH. The maximum adsorption of I^−^ onto the GO/CS sponges was found at pH 7.2 (q_e_ = 32.1 mg/g), where I^−^ could create a bond between the positively charged surface of GO without the effect of H^+^ and OH^−^. When the pH value increased, the surface charge of GO was fundamentally negative; this may have led to the decrease in the adsorption capacity of GO/CS sponges [28]. 

**Figure 12:**
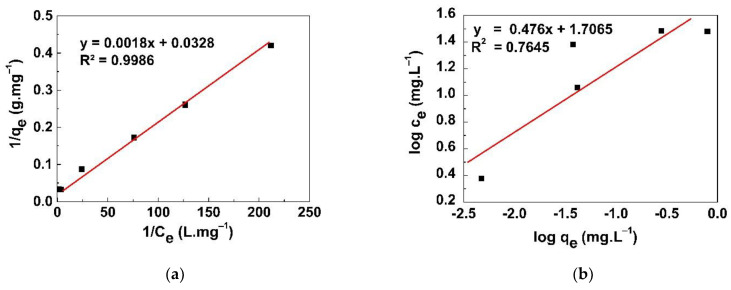
(**a**) Langmuir and (**b**) Freundlich adsorption isotherms for iodide removal at various concentrations.

Adsorption isotherms are very useful for demonstrating the amount of material adsorbed per unit of mass of the adsorbent as a function of the equilibrium concentration of the adsorbate. The Langmuir and Freundlich adsorption isotherms for iodide removal from aqueous solutions by the GO/CS sponges are shown in Figure 12a,b, respectively. In the case of the Langmuir isotherm model, the linear plot between 1/q_e_ and 1/C_e_ resulted in a slope of 1/(q_max_·K_L_) and an intercept of 1/q_max_. For the Freundlich model, a graph between log C_e_ and log q_e_ resulted in a slope of 1/n and an intercept of log K_F_ [29]. The Langmuir and Freundlich isotherm parameters calculated from the slopes and intercepts of the linear plots are presented in Table 1. The maximum adsorption capacity was found to be 30.5 mg/g, and the Langmuir constant (K_L_) was 0.0182 L/mg. In order to describe the affinity between the adsorbent and adsorbate, the Langmuir isotherm can be expressed by a dimensionless constant called the separation factor (R_L_) [30], as shown in Equation (6):

(6) $$R_{L}=\frac{1}{1+K_{L}C_{o}}$$

where *C_o_* is the initial concentration of iodide ions and *K_L_* is the Langmuir constant. The *R_L_* value indicates the shape of the isotherm if it is consistent with the adsorption. A value of *R_L_* > 1 indicates unfavorable uptake, *R_L_* = 1 indicates linear uptake, 0 < *R_L_* < 1 indicates favorable uptake, and *R_L_* = 0 indicates irreversible uptake [31]. The *R_L_* value (0.6295) observed in our study is in the range of 0–1, confirming the favorable uptake of the iodide by the GO/CS sponges. Furthermore, the correlation coefficients (R^2^) of the Langmuir and Freundlich isotherms were found to be 0.9986 and 0.7645, respectively, demonstrating that the Langmuir isotherm completely conformed to the experimental data. This finding confirmed that the GO/CS sponges’ structure was uniform monolayer GO sheets. 

In light of the Langmuir isotherm parameters derived from our experiments, they can be used to determine the adsorption capacity of GO/CS sponges to remove radioactive iodine-131 from wastewater. As iodine-131 is classified as a carrier-free radioisotope, its specific activity can be determined by the equation A = λN, where A is radioactivity (Bq), λ is the decay constant (where λ = 0.693/T_1/2_), and N is the number of radioactive atoms [32]. The maximum adsorption for iodine-131 calculated from the Langmuir isotherm parameters is 1.1927 × 10^8^ MBq/g. 

**Figure 13:**
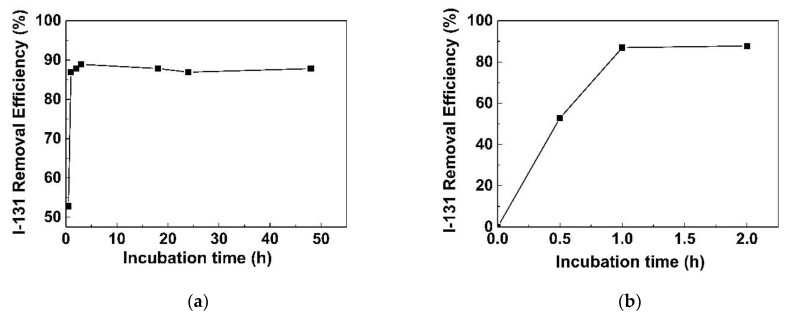
The removal efficiency of I-131 by GO/CS at different incubation times: (**a**) 0.5 h to 48 h and (**b**) 0.5 h to 2 h.

In order to determine the efficiency of the synthesized GO/CS sponges for the removal of radioactive iodine from wastewater, adsorption experiments were conducted by varying the incubation time and the weight of the GO/CS sponges. The effects of the incubation period on adsorption capacity are shown in Figure 13. It is apparent that adsorption was rapid in the first 1 h but then slowed until reaching equilibrium. In the initial stage, the high removal rate was probably due to the rapid contact of sodium iodide molecules with the active sites on the external surfaces of the GO/CS sponge adsorbent. The subsequently constant rate might be attributed to the diminishing availability of the remaining active sites. However, a 24 h incubation period was chosen in our study to guarantee sufficient adsorption.

**Figure 14:**
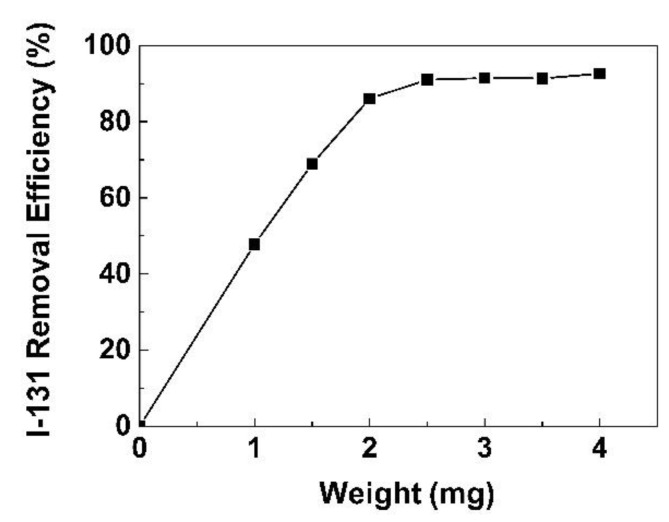
The removal efficiency of iodine-131 by GO/CS sponges at 24 h.

Figure 14 shows the removal efficiency of iodine-131 by GO/CS sponges calculated by Equations (4) and (5). The removal efficiency increased with an increase in the weight of the GO/CS sponges from 1.0 to 2.0 mg. After that, the removal efficiency was relatively constant. At a weight of 1.5 mg, the removal efficiency was 68.9%. The highest removal efficiency was found at 92.6% for the weight of 4.0 mg, which is equivalent to 0.263 MBq iodine-131 per milligram of adsorbent at pH 7.2 ± 0.2 for 24 h of adsorption. Based on the release of iodine-131 demonstrated by Larsen et al. [33], a treatment dose of ~3.8 GBq (102.6 mCi) of iodine-131 in a patient at a local hospital resulted in iodine-131 releases of approximately 3.2 GBq (87 mCi) entering the sewer system over a 40 h period. In comparison with those observations, the synthesized GO/CS demonstrated reasonable adsorbing capacity, i.e., 12.167 g of GO/CS was needed to remove 3.2 GBq. Since the cost of GO/CS is relatively cheap compared with various commercially available adsorbents, it would be possible to replace the costly adsorbents with the GO/CS sponges developed here. 

When the GO/CS sponges were compared with conventional adsorbents such as activated carbon, although GO/CS sponges are more difficult to synthesize than activated carbon, our study showed that the synthesized GO/CS sponges were quite effective. Iodine can be absorbed to the maximum adsorption capacity in as short as 1 h, whereas activated carbon has a very slow adsorption time of 48 h [34]. The distinguishing feature of the GO/CS sponges is that they are easily shaped; therefore, they are convenient for filtering or trapping iodine at specific points such as in hospital sewer systems. Another interesting point of the GO/CS sponges is their handling after iodine adsorption in the solution. They can be easily separated from the solution by means of a clamping device. Because of the physical nature of the GO/CS sponges, in which the graphene oxide sheets are connected to chitosan, they are not easily broken. Unlike activated carbon, which is a fine powder mixed with a solution, filtration is required to separate them. In addition, our study showed that the developed GO/CS sponges adsorbed iodine-131 at a high efficiency of 90% within 1 h without external energy use. This finding is interesting because radiation containers can be placed under radiation-shielding materials. This supports the best practices of working with radiation: ALARA (“As Low as Reasonably Achievable”), which means that all reasonable effort is needed to keep radiation exposure below the radiation dose limit as much as possible. 

Among the many advantages of the GO/CS sponges, they can be shaped in a wide variety of containers used in the freeze-drying process and the sponges do not dissolve into the water; thus, the synthesized sorbent can be used in practical applications. For example, they may be added to a column to adsorb iodine-131 from pouring wastes, which can significantly reduce the amount of liquid radioactive waste that must be stored until being drained. These advantages not only reduce the cost of the waste storage system but also reduce storage space, as the secondary wastes arising from sorbents are several times smaller than liquid wastes. Due to the adsorption efficiency of the sorbent (12.167 g sorbent required to remove 3.12 GBq), the amount of solid secondary waste generated would be extremely low and can be stored in a small lead-shielded container until decay. We expect to explore the renewable performance of adsorbents further. This would greatly minimize the secondary waste generated. In addition, the sorbent will be particularly useful for increasing the number of patients receiving radioiodine therapy, as it can speed up the treatment time of contaminated liquid waste. Most importantly, the waste management system to be built for a new hospital does not have to be at a large scale that would normally be required for basic radioactive waste treatment principles: (1) concentrate and contain (2) delay and decay, and (3) dilute and disperse.

## 4. Conclusions

We successfully synthesized graphene oxide/chitosan (GO/CS) sponges as an adsorbent for the removal of iodine-131 from aqueous solutions. The adsorption isotherm experiment was consistent with Langmuir’s isotherm model, which indicates that the synthetic structure consisted of randomly connected GO sheets without overlapping layers. Our study demonstrated that the synthesized GO/CS sponges could effectively adsorb radioactive iodine-131 in aqueous solution at pH 7.2 ± 0.2. The maximum adsorption capacity of iodine-131 by GO/CS was 0.263 MBq/mg. Based on the information of iodine-131 released from patients undergoing therapeutic application and entering a municipal sewer, the amount of GO/CS needed for radioactive iodide treatment is feasible. Due to its attractive characteristics, including the low cost, the ease of obtaining it, and its eco-friendly properties, the GO/CS developed here could be used as an alternative adsorbent for removing radioiodine from wastewater. It may be applied to adsorb liquid iodine-131 wastes from wards before releasing them into the sewage waste drainage system.

## Figures and Tables

**Table 1:** Langmuir and Freundlich isotherm parameters for adsorption of iodide ions from a solution by GO/CS sponges.

Langmuir Isotherm Model				Freundlich Isotherm Model		
q_max_ (mg/g)	K_L_ (L/mg)	R^2^	R_L_	K_F_ (mg/g)	1/n	R^2^
30.4878	0.0182	0.9986	0.6295	50.8745	0.4760	0.7645

## Data Availability

Not applicable.
